# {[(1*Z*)-3-Chloro-1*H*-isoindol-1-yl­idene]meth­yl}dimethyl­amine

**DOI:** 10.1107/S160053681300500X

**Published:** 2013-02-28

**Authors:** Zhao-Yun Wang, Chang-Jiang Yu

**Affiliations:** a School of Chemistry and Material Science, Anhui Normal University, Wuhu, People’s Republic of China

## Abstract

The asymmetric unit of the title compound, C_11_H_11_ClN_2_, contains two almost-planar independent mol­ecules: the isoindole and dimethyl­amino­methyl­ene mean planes in the two mol­ecules form dihedral angles of 5.45 (8) and 1.34 (8)°. The crystal packing exhibits no short inter­molecular contacts, except for a relatively short Cl⋯Cl distance of 3.4907 (7) Å.

## Related literature

For applications of related isoindole derivatives, see: Jiao *et al.* (2010 ▶). For details of the synthesis, see: von Doheneck *et al.* (1969 ▶). For the crystal structure of the related compound 4,5,6,7-tetra­fluoro-1-(*N*,*N*-dimethyl­amino­methyl­ene)-1*H*-isoindole, see: Uno *et al.* (2007 ▶).
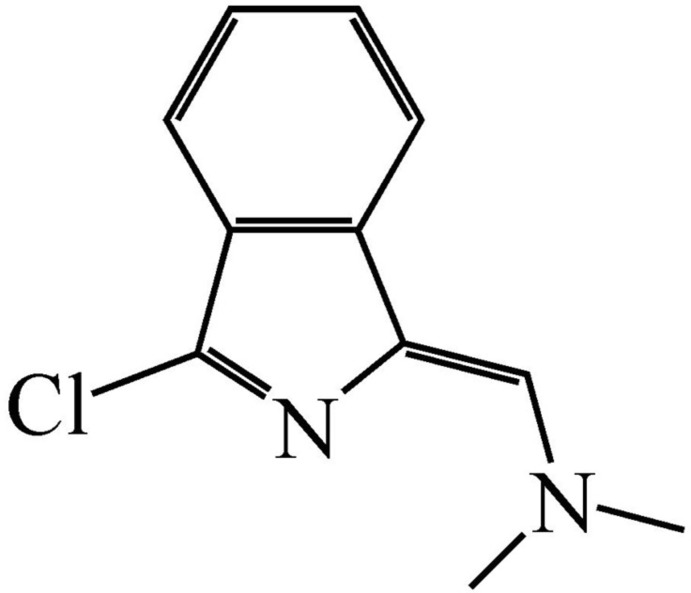


## Experimental

### 

#### Crystal data

C_11_H_11_ClN_2_
*M* *_r_* = 206.67
Monoclinic, 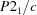

*a* = 12.1588 (9) Å
*b* = 10.4087 (8) Å
*c* = 16.3165 (12) Å
β = 92.720 (1)°
*V* = 2062.6 (3) Å^3^
*Z* = 8
Mo *K*α radiation
μ = 0.33 mm^−1^
*T* = 293 K
0.30 × 0.30 × 0.20 mm

#### Data collection

Bruker SMART APEX CCD diffractometer
Absorption correction: multi-scan (*SADABS*; Bruker, 2002 ▶) *T*_min_ = 0.908, *T*_max_ = 0.937
14393 measured reflections
3628 independent reflections
3175 reflections with *I* > 2σ(*I*)
*R*_int_ = 0.023

#### Refinement

*R*[*F* ^2^ > 2σ(*F* ^2^)] = 0.032
*wR*(*F* ^2^) = 0.092
*S* = 1.02
3628 reflections
257 parameters
H-atom parameters constrained
Δρ_max_ = 0.22 e Å^−3^
Δρ_min_ = −0.25 e Å^−3^

### 

Data collection: *SMART* (Bruker, 2002 ▶); cell refinement: *SAINT* (Bruker, 2002 ▶); data reduction: *SAINT*; program(s) used to solve structure: *SHELXS97* (Sheldrick, 2008 ▶); program(s) used to refine structure: *SHELXL97* (Sheldrick, 2008 ▶); molecular graphics: *SHELXTL* (Sheldrick, 2008 ▶); software used to prepare material for publication: *SHELXTL*.

## Supplementary Material

Crystal structure: contains datablock(s) I, global. DOI: 10.1107/S160053681300500X/cv5388sup1.cif

Structure factors: contains datablock(s) I. DOI: 10.1107/S160053681300500X/cv5388Isup2.hkl

Supplementary material file. DOI: 10.1107/S160053681300500X/cv5388Isup3.cml

Additional supplementary materials:  crystallographic information; 3D view; checkCIF report

## Figures and Tables

**Fig. 1.**
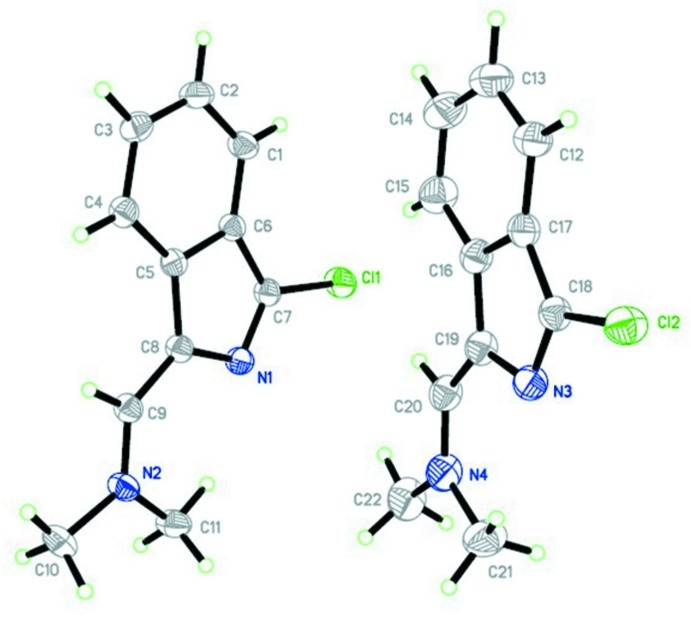
Two independent molecules of (I) showing the atomic numbering and 30% probability displacement ellipsoids.

## supplementary crystallographic information

### Comment

The title compound, (I), was obtained as an intermediate in our ongoing search (Jiao *et al.*, 2010) for new synthetic routes to boron-dipyrromethene (BODIPY). In (I) (Fig. 1), the bond lengths and angles are normal and correspond to those observed in the related 4,5,6,7-tetrafluoro-1-(N,N-dimethylaminomethylene)-1H-isoindole (Uno *et al.*, 2007). In the crystal structure, there are no short intermolecular contacts, except of relatively short Cl···Cl distance of 3.4907 (7) Å.

### Experimental

The title compound was prepared following the known procedure (von Doheneck *et al.*, 1969). To DMF (2.4 ml, 30 mmol) in 7 ml CH_2_Cl_2_ was added POCl_3_ (2.0 ml, 20 mmol), the reaction mixture was stirred under ice-cold condition for 0.5 h. Then isoindolin-1-one (1 g, 7.5 mmol) in 10 ml CH_2_Cl_2_ was added. After stirring at 333 k for 3 h, the reaction was monitored by TLC, adjusted pH-value to 8 with saturated potassium carbonate, poured into water (50 ml) and extracted with CH_2_Cl_2_ (3\30 ml). Organic layers were combined, dried over Na_2_SO_4_, and evaporated to dryness under vacuum. The desired compound was obtained as powder in 30% (280 mg) from column chromatography.

### Refinement

All hydrogen atoms were placed in geometrically idealized positions and constrained to ride on their parent atoms, with C—H = 0.93 - 0.97 Å and *U*_iso_(H) = 1.2 - 1.5 *U*_eq_.

### Crystal data

**Table:**

C_11_H_11_ClN_2_	*F*(000) = 864
*M**_r_* = 206.67	*D*_x_ = 1.331 Mg m^−^^3^
Monoclinic, *P*2_1_/*c*	Mo *K*α radiation, λ = 0.71073 Å
Hall symbol: -P 2ybc	Cell parameters from 9633 reflections
*a* = 12.1588 (9) Å	θ = 2.3–27.6°
*b* = 10.4087 (8) Å	µ = 0.33 mm^−^^1^
*c* = 16.3165 (12) Å	*T* = 293 K
β = 92.720 (1)°	Block, colourless
*V* = 2062.6 (3) Å^3^	0.30 × 0.30 × 0.20 mm
*Z* = 8	

### Data collection

**Table:**

Bruker SMART APEX CCD diffractometer	3628 independent reflections
Radiation source: fine-focus sealed tube	3175 reflections with *I* > 2σ(*I*)
Graphite monochromator	*R*_int_ = 0.023
phi and ω scans	θ_max_ = 25.0°, θ_min_ = 1.7°
Absorption correction: multi-scan (*SADABS*; Bruker, 2002)	*h* = −12→14
*T*_min_ = 0.908, *T*_max_ = 0.937	*k* = −12→12
14393 measured reflections	*l* = −19→19

### Refinement

**Table:**

Refinement on *F*^2^	Primary atom site location: structure-invariant direct methods
Least-squares matrix: full	Secondary atom site location: difference Fourier map
*R*[*F*^2^ > 2σ(*F*^2^)] = 0.032	Hydrogen site location: inferred from neighbouring sites
*wR*(*F*^2^) = 0.092	H-atom parameters constrained
*S* = 1.02	*w* = 1/[σ^2^(*F*_o_^2^) + (0.0482*P*)^2^ + 0.6549*P*] where *P* = (*F*_o_^2^ + 2*F*_c_^2^)/3
3628 reflections	(Δ/σ)_max_ = 0.001
257 parameters	Δρ_max_ = 0.22 e Å^−^^3^
0 restraints	Δρ_min_ = −0.25 e Å^−^^3^

### Special details

**Table:**

Geometry. All e.s.d.'s (except the e.s.d. in the dihedral angle between two l.s. planes) are estimated using the full covariance matrix. The cell e.s.d.'s are taken into account individually in the estimation of e.s.d.'s in distances, angles and torsion angles; correlations between e.s.d.'s in cell parameters are only used when they are defined by crystal symmetry. An approximate (isotropic) treatment of cell e.s.d.'s is used for estimating e.s.d.'s involving l.s. planes.
Refinement. Refinement of *F*^2^ against ALL reflections. The weighted *R*-factor *wR* and goodness of fit *S* are based on *F*^2^, conventional *R*-factors *R* are based on *F*, with *F* set to zero for negative *F*^2^. The threshold expression of *F*^2^ > σ(*F*^2^) is used only for calculating *R*-factors(gt) *etc*. and is not relevant to the choice of reflections for refinement. *R*-factors based on *F*^2^ are statistically about twice as large as those based on *F*, and *R*- factors based on ALL data will be even larger.

### Fractional atomic coordinates and isotropic or equivalent isotropic displacement parameters (Å^2^)

**Table:**

	*x*	*y*	*z*	*U*_iso_*/*U*_eq_	
C1	0.10344 (14)	0.95021 (15)	0.17664 (10)	0.0446 (4)	
H1	0.0995	1.0308	0.1520	0.054*	
C2	0.09492 (15)	0.93661 (16)	0.25997 (10)	0.0485 (4)	
H2	0.0855	1.0090	0.2923	0.058*	
C3	0.10025 (14)	0.81523 (16)	0.29649 (10)	0.0462 (4)	
H3	0.0948	0.8086	0.3530	0.055*	
C4	0.11330 (13)	0.70544 (15)	0.25124 (9)	0.0413 (4)	
H4	0.1158	0.6253	0.2764	0.050*	
C5	0.12269 (12)	0.71688 (14)	0.16626 (9)	0.0351 (3)	
C6	0.11816 (12)	0.83963 (14)	0.13021 (9)	0.0360 (3)	
C7	0.13229 (13)	0.81558 (14)	0.04517 (9)	0.0379 (3)	
C8	0.13736 (12)	0.62763 (14)	0.09977 (9)	0.0363 (3)	
C9	0.14062 (12)	0.49581 (14)	0.10851 (9)	0.0382 (3)	
H9	0.1383	0.4670	0.1624	0.046*	
C10	0.14543 (16)	0.26882 (15)	0.07727 (12)	0.0540 (4)	
H10A	0.1493	0.2628	0.1360	0.081*	
H10B	0.2076	0.2257	0.0557	0.081*	
H10C	0.0787	0.2292	0.0560	0.081*	
C11	0.14694 (16)	0.42637 (17)	−0.03484 (10)	0.0521 (4)	
H11A	0.1402	0.5168	−0.0454	0.078*	
H11B	0.0862	0.3819	−0.0618	0.078*	
H11C	0.2147	0.3955	−0.0554	0.078*	
C12	0.59631 (16)	1.03344 (17)	0.23675 (11)	0.0538 (4)	
H12	0.6607	1.0464	0.2688	0.065*	
C13	0.50202 (19)	1.1010 (2)	0.25156 (13)	0.0660 (5)	
H13	0.5023	1.1603	0.2942	0.079*	
C14	0.40623 (19)	1.0810 (2)	0.20311 (14)	0.0709 (6)	
H14	0.3431	1.1266	0.2149	0.085*	
C15	0.40161 (16)	0.9966 (2)	0.13874 (13)	0.0624 (5)	
H15	0.3371	0.9864	0.1065	0.075*	
C16	0.49652 (14)	0.92593 (16)	0.12242 (10)	0.0478 (4)	
C17	0.59264 (14)	0.94515 (15)	0.17246 (10)	0.0448 (4)	
C18	0.66982 (13)	0.85782 (16)	0.13977 (10)	0.0434 (4)	
C19	0.52280 (14)	0.82944 (16)	0.06357 (10)	0.0471 (4)	
C20	0.45005 (15)	0.78325 (18)	0.00284 (11)	0.0528 (4)	
H20	0.3807	0.8210	0.0022	0.063*	
C21	0.56360 (18)	0.6267 (2)	−0.06552 (14)	0.0758 (6)	
H21A	0.6006	0.6639	−0.1105	0.114*	
H21B	0.5476	0.5381	−0.0772	0.114*	
H21C	0.6100	0.6327	−0.0164	0.114*	
C22	0.37124 (18)	0.6668 (2)	−0.11346 (14)	0.0765 (7)	
H22A	0.3079	0.7170	−0.1011	0.115*	
H22B	0.3533	0.5771	−0.1107	0.115*	
H22C	0.3931	0.6873	−0.1677	0.115*	
Cl1	0.13909 (4)	0.93494 (4)	−0.02823 (2)	0.05378 (15)	
Cl2	0.80400 (4)	0.83930 (5)	0.17864 (3)	0.05637 (15)	
N1	0.14301 (11)	0.69544 (12)	0.02584 (8)	0.0394 (3)	
N2	0.14649 (11)	0.40346 (12)	0.05304 (8)	0.0425 (3)	
N3	0.63250 (11)	0.79025 (14)	0.07737 (8)	0.0470 (3)	
N4	0.46144 (13)	0.69555 (17)	−0.05409 (10)	0.0597 (4)	

### Atomic displacement parameters (Å^2^)

**Table:**

	*U*^11^	*U*^22^	*U*^33^	*U*^12^	*U*^13^	*U*^23^
C1	0.0570 (10)	0.0325 (8)	0.0444 (9)	0.0018 (7)	0.0031 (7)	0.0002 (6)
C2	0.0598 (11)	0.0440 (9)	0.0418 (9)	0.0026 (8)	0.0044 (8)	−0.0093 (7)
C3	0.0534 (10)	0.0532 (10)	0.0322 (8)	−0.0002 (8)	0.0028 (7)	−0.0016 (7)
C4	0.0485 (9)	0.0403 (8)	0.0351 (8)	−0.0013 (7)	0.0000 (7)	0.0056 (6)
C5	0.0352 (8)	0.0344 (7)	0.0356 (8)	−0.0012 (6)	0.0001 (6)	0.0017 (6)
C6	0.0387 (8)	0.0338 (7)	0.0353 (8)	−0.0008 (6)	0.0011 (6)	0.0020 (6)
C7	0.0461 (9)	0.0338 (8)	0.0339 (8)	0.0001 (6)	0.0020 (6)	0.0053 (6)
C8	0.0426 (8)	0.0330 (7)	0.0330 (7)	0.0004 (6)	−0.0005 (6)	0.0029 (6)
C9	0.0422 (8)	0.0359 (8)	0.0363 (8)	0.0003 (6)	−0.0007 (6)	0.0032 (6)
C10	0.0635 (11)	0.0305 (8)	0.0678 (12)	0.0005 (8)	−0.0006 (9)	0.0020 (8)
C11	0.0700 (12)	0.0432 (9)	0.0431 (9)	0.0055 (8)	0.0020 (8)	−0.0062 (7)
C12	0.0610 (11)	0.0499 (10)	0.0507 (10)	0.0000 (8)	0.0022 (8)	−0.0042 (8)
C13	0.0795 (14)	0.0571 (12)	0.0621 (12)	0.0092 (10)	0.0101 (10)	−0.0101 (9)
C14	0.0653 (13)	0.0690 (13)	0.0794 (15)	0.0198 (10)	0.0122 (11)	−0.0055 (11)
C15	0.0512 (11)	0.0667 (12)	0.0692 (12)	0.0101 (9)	0.0008 (9)	−0.0007 (10)
C16	0.0481 (9)	0.0486 (9)	0.0468 (9)	0.0005 (7)	0.0038 (7)	0.0047 (7)
C17	0.0501 (9)	0.0416 (9)	0.0429 (9)	−0.0018 (7)	0.0047 (7)	0.0043 (7)
C18	0.0429 (9)	0.0456 (9)	0.0415 (9)	−0.0034 (7)	0.0004 (7)	0.0006 (7)
C19	0.0431 (9)	0.0531 (10)	0.0450 (9)	−0.0012 (7)	0.0005 (7)	0.0016 (7)
C20	0.0452 (10)	0.0637 (11)	0.0493 (10)	−0.0022 (8)	0.0007 (8)	0.0013 (9)
C21	0.0614 (13)	0.0909 (16)	0.0746 (14)	−0.0004 (11)	−0.0014 (11)	−0.0300 (13)
C22	0.0618 (13)	0.1050 (19)	0.0612 (13)	−0.0195 (12)	−0.0116 (10)	−0.0111 (12)
Cl1	0.0843 (3)	0.0378 (2)	0.0396 (2)	0.00008 (19)	0.0071 (2)	0.01022 (16)
Cl2	0.0481 (3)	0.0647 (3)	0.0553 (3)	0.0043 (2)	−0.00815 (19)	−0.0132 (2)
N1	0.0484 (7)	0.0355 (7)	0.0342 (7)	0.0003 (6)	0.0030 (6)	0.0010 (5)
N2	0.0530 (8)	0.0308 (6)	0.0436 (7)	0.0030 (6)	−0.0001 (6)	0.0003 (6)
N3	0.0453 (8)	0.0512 (8)	0.0445 (8)	−0.0018 (6)	0.0009 (6)	−0.0026 (6)
N4	0.0486 (9)	0.0767 (11)	0.0533 (9)	−0.0088 (8)	−0.0045 (7)	−0.0110 (8)

### Geometric parameters (Å, º)

**Table:**

C1—C2	1.376 (2)	C12—C13	1.376 (3)
C1—C6	1.394 (2)	C12—C17	1.394 (2)
C1—H1	0.9300	C12—H12	0.9300
C2—C3	1.397 (2)	C13—C14	1.392 (3)
C2—H2	0.9300	C13—H13	0.9300
C3—C4	1.374 (2)	C14—C15	1.368 (3)
C3—H3	0.9300	C14—H14	0.9300
C4—C5	1.402 (2)	C15—C16	1.405 (3)
C4—H4	0.9300	C15—H15	0.9300
C5—C6	1.406 (2)	C16—C17	1.408 (2)
C5—C8	1.446 (2)	C16—C19	1.436 (2)
C6—C7	1.428 (2)	C17—C18	1.428 (2)
C7—N1	1.2977 (19)	C18—N3	1.301 (2)
C7—Cl1	1.7303 (14)	C18—Cl2	1.7328 (16)
C8—C9	1.380 (2)	C19—C20	1.383 (2)
C8—N1	1.4021 (19)	C19—N3	1.403 (2)
C9—N2	1.325 (2)	C20—N4	1.314 (2)
C9—H9	0.9300	C20—H20	0.9300
C10—N2	1.456 (2)	C21—N4	1.454 (3)
C10—H10A	0.9600	C21—H21A	0.9600
C10—H10B	0.9600	C21—H21B	0.9600
C10—H10C	0.9600	C21—H21C	0.9600
C11—N2	1.454 (2)	C22—N4	1.460 (2)
C11—H11A	0.9600	C22—H22A	0.9600
C11—H11B	0.9600	C22—H22B	0.9600
C11—H11C	0.9600	C22—H22C	0.9600
			
C2—C1—C6	118.01 (14)	C12—C13—H13	119.8
C2—C1—H1	121.0	C14—C13—H13	119.8
C6—C1—H1	121.0	C15—C14—C13	122.32 (19)
C1—C2—C3	120.69 (15)	C15—C14—H14	118.8
C1—C2—H2	119.7	C13—C14—H14	118.8
C3—C2—H2	119.7	C14—C15—C16	118.53 (19)
C4—C3—C2	121.82 (15)	C14—C15—H15	120.7
C4—C3—H3	119.1	C16—C15—H15	120.7
C2—C3—H3	119.1	C15—C16—C17	118.84 (17)
C3—C4—C5	118.54 (14)	C15—C16—C19	134.76 (17)
C3—C4—H4	120.7	C17—C16—C19	106.39 (15)
C5—C4—H4	120.7	C12—C17—C16	121.75 (16)
C4—C5—C6	119.17 (14)	C12—C17—C18	134.65 (17)
C4—C5—C8	134.94 (14)	C16—C17—C18	103.60 (14)
C6—C5—C8	105.89 (13)	N3—C18—C17	115.17 (15)
C1—C6—C5	121.76 (14)	N3—C18—Cl2	120.90 (13)
C1—C6—C7	134.18 (14)	C17—C18—Cl2	123.93 (13)
C5—C6—C7	104.06 (13)	C20—C19—N3	125.80 (16)
N1—C7—C6	115.03 (13)	C20—C19—C16	124.55 (16)
N1—C7—Cl1	121.00 (12)	N3—C19—C16	109.65 (14)
C6—C7—Cl1	123.95 (11)	N4—C20—C19	131.49 (18)
C9—C8—N1	125.96 (14)	N4—C20—H20	114.3
C9—C8—C5	124.43 (13)	C19—C20—H20	114.3
N1—C8—C5	109.56 (12)	N4—C21—H21A	109.5
N2—C9—C8	130.78 (14)	N4—C21—H21B	109.5
N2—C9—H9	114.6	H21A—C21—H21B	109.5
C8—C9—H9	114.6	N4—C21—H21C	109.5
N2—C10—H10A	109.5	H21A—C21—H21C	109.5
N2—C10—H10B	109.5	H21B—C21—H21C	109.5
H10A—C10—H10B	109.5	N4—C22—H22A	109.5
N2—C10—H10C	109.5	N4—C22—H22B	109.5
H10A—C10—H10C	109.5	H22A—C22—H22B	109.5
H10B—C10—H10C	109.5	N4—C22—H22C	109.5
N2—C11—H11A	109.5	H22A—C22—H22C	109.5
N2—C11—H11B	109.5	H22B—C22—H22C	109.5
H11A—C11—H11B	109.5	C7—N1—C8	105.46 (12)
N2—C11—H11C	109.5	C9—N2—C11	123.90 (13)
H11A—C11—H11C	109.5	C9—N2—C10	120.76 (14)
H11B—C11—H11C	109.5	C11—N2—C10	115.21 (13)
C13—C12—C17	118.16 (18)	C18—N3—C19	105.19 (14)
C13—C12—H12	120.9	C20—N4—C21	123.54 (16)
C17—C12—H12	120.9	C20—N4—C22	120.70 (18)
C12—C13—C14	120.38 (19)	C21—N4—C22	115.68 (17)
			
C6—C1—C2—C3	−0.4 (3)	C13—C12—C17—C18	−178.64 (19)
C1—C2—C3—C4	−0.5 (3)	C15—C16—C17—C12	−0.9 (3)
C2—C3—C4—C5	0.7 (3)	C19—C16—C17—C12	179.98 (15)
C3—C4—C5—C6	−0.2 (2)	C15—C16—C17—C18	178.96 (16)
C3—C4—C5—C8	179.67 (17)	C19—C16—C17—C18	−0.17 (17)
C2—C1—C6—C5	1.0 (2)	C12—C17—C18—N3	180.00 (18)
C2—C1—C6—C7	−178.50 (17)	C16—C17—C18—N3	0.18 (19)
C4—C5—C6—C1	−0.7 (2)	C12—C17—C18—Cl2	0.3 (3)
C8—C5—C6—C1	179.44 (14)	C16—C17—C18—Cl2	−179.51 (12)
C4—C5—C6—C7	178.93 (14)	C15—C16—C19—C20	1.2 (3)
C8—C5—C6—C7	−0.97 (16)	C17—C16—C19—C20	−179.85 (17)
C1—C6—C7—N1	−179.57 (17)	C15—C16—C19—N3	−178.8 (2)
C5—C6—C7—N1	0.91 (19)	C17—C16—C19—N3	0.13 (19)
C1—C6—C7—Cl1	2.2 (3)	N3—C19—C20—N4	0.8 (3)
C5—C6—C7—Cl1	−177.28 (12)	C16—C19—C20—N4	−179.20 (19)
C4—C5—C8—C9	3.3 (3)	C6—C7—N1—C8	−0.42 (19)
C6—C5—C8—C9	−176.81 (15)	Cl1—C7—N1—C8	177.84 (11)
C4—C5—C8—N1	−179.07 (16)	C9—C8—N1—C7	177.31 (16)
C6—C5—C8—N1	0.80 (17)	C5—C8—N1—C7	−0.25 (17)
N1—C8—C9—N2	−2.0 (3)	C8—C9—N2—C11	−2.3 (3)
C5—C8—C9—N2	175.21 (15)	C8—C9—N2—C10	−178.06 (16)
C17—C12—C13—C14	−0.1 (3)	C17—C18—N3—C19	−0.11 (19)
C12—C13—C14—C15	−1.2 (4)	Cl2—C18—N3—C19	179.60 (12)
C13—C14—C15—C16	1.4 (3)	C20—C19—N3—C18	179.96 (17)
C14—C15—C16—C17	−0.4 (3)	C16—C19—N3—C18	−0.02 (19)
C14—C15—C16—C19	178.4 (2)	C19—C20—N4—C21	−1.5 (3)
C13—C12—C17—C16	1.2 (3)	C19—C20—N4—C22	−178.1 (2)
